# Evolution of the tripartite symbiosis between earthworms, *Verminephrobacter* and *Flexibacter*-like bacteria

**DOI:** 10.3389/fmicb.2015.00529

**Published:** 2015-05-27

**Authors:** Peter Møller, Marie B. Lund, Andreas Schramm

**Affiliations:** ^1^Section for Microbiology, Department of Bioscience, Aarhus UniversityAarhus, Denmark; ^2^Aarhus Institute of Advanced Studies, Aarhus UniversityAarhus, Denmark

**Keywords:** co-evolution, co-speciation, symbiosis, *Verminephrobacter*, *Flexibacter*-like, *Ca*. Nephrothrix, earthworm, nephridia

## Abstract

Nephridial (excretory organ) symbionts are widespread in lumbricid earthworms and the complexity of the nephridial symbiont communities varies greatly between earthworm species. The two most common symbionts are the well-described *Verminephrobacter* and less well-known *Flexibacter*-like bacteria. *Verminephrobacter* are present in almost all lumbricid earthworms, they are species-specific, vertically transmitted, and have presumably been associated with their hosts since the origin of lumbricids. *Flexibacter*-like symbionts have been reported from about half the investigated earthworms; they are also vertically transmitted. To investigate the evolution of this tri-partite symbiosis, phylogenies for 18 lumbricid earthworm species were constructed based on two mitochondrial genes, NADH dehydrogenase subunit 2 (ND2) and cytochrome c oxidase subunit I (COI), and compared to their symbiont phylogenies based on RNA polymerase subunit B (*rpoB*) and 16S rRNA genes. The two nephridial symbionts showed markedly different evolutionary histories with their hosts. For *Verminephrobacter*, clear signs of long-term host-symbiont co-evolution with rare host switching events confirmed its ancient association with lumbricid earthworms, likely dating back to their last common ancestor about 100 million years (MY) ago. In contrast, phylogenies for the *Flexibacter*-like symbionts suggested an ability to switch to new hosts, to which they adapted and subsequently became species-specific. Putative co-speciation events were only observed with closely related host species; on that basis, this secondary symbiosis was estimated to be minimum 45 MY old. Based on the monophyletic clustering of the *Flexibacter*-like symbionts, the low 16S rRNA gene sequence similarity to the nearest described species (<92%) and environmental sequences (<94.2%), and the specific habitat in the earthworm nephridia, we propose a new candidate genus for this group, *Candidatus* Nephrothrix.

## Introduction

**Figure 1:**
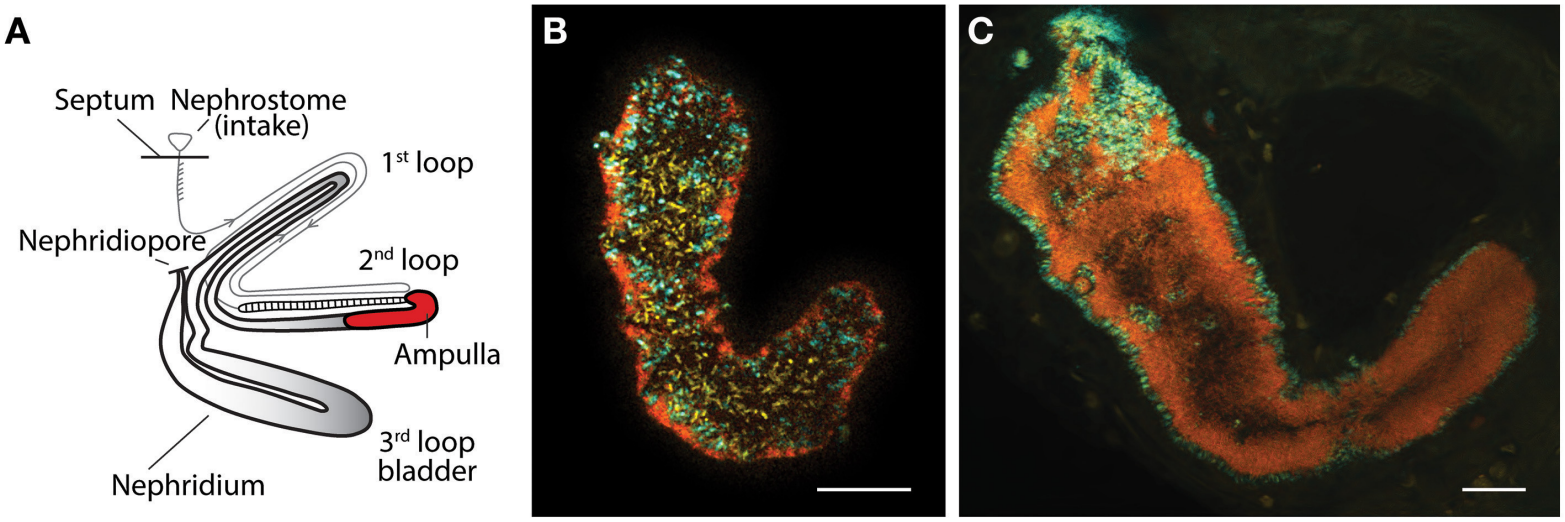
**(A)** Schematic outline of the structure of a single nephridium showing the three major loops and the ampulla (red) where the symbionts reside (modified from Schramm et al., 2003). **(B,C)** FISH images of ampullas from two earthworm species after triple hybridization with the general bacterial probe EUB338,-II,-III (yellow), a *Verminephrobacter*-specific probe LSB145 (cyan/green), and a probe specific for *Flexibacter*-like symbionts FLX-226 (red/orange). **(B)** *Al. chlorotica* showing the *Flexibacter*-like symbionts in close proximity to the ampulla epithelium and *Verminephrobacter* in the lumen. Notice the large amount of unidentified bacteria in the lumen. **(C)** *E. andrei* with *Verminephrobacter* lining the epithelium and the *Flexibacter*-like symbionts in the lumen. Scale bar is 20 μm.

Earthworms of the family Lumbricidae have long been known to harbor extracellular symbiotic bacteria in their nephridia (Knop, 1926). The nephridia are the worm's excretory system involved in excretion of nitrogenous waste and osmoregulation (Edwards and Bohlen, 1996). They are found in pairs in every segment, and each nephridium is coiled into three loops, where the symbionts reside in the ampulla part of the second loop (Figure 1A). Complexity and composition of the symbiont communities vary between worm species; few species host exclusively the betaproteobacterial *Verminephrobacter*, while most lumbricids have mixed nephridial communities (Davidson et al., 2010, 2013; Lund et al., 2010a). *Verminephrobacter* have been studied in detail during the past decade (for a recent review see Lund et al., 2014); these symbionts are species-specific (Schramm et al., 2003; Lund et al., 2010a), beneficial (Lund et al., 2010b), vertically transmitted via the cocoon (Davidson and Stahl, 2006, 2008), and presumably co-evolved with their host (Lund et al., 2010a).

In contrast, little is known about the other nephridial symbionts that represent at least 27 different taxa from six phyla and occur inconsistently across earthworm species (Davidson et al., 2013). Most information is available for a *Flexibacter*-like symbiont (phylum Bacteroidetes), which was first discovered in the compost worm *Eisenia fetida* and shown to be vertically transmitted via the cocoon, together with *Verminephrobacter* and a member of the Microbacteriaceae (Davidson et al., 2010). The *Flexibacter-*like symbionts even seem to colonize the ampulla of developing embryos before the arrival of *Verminephrobacter* (Davidson et al., 2010; Dulla et al., 2012). In a global survey, *Flexibacter-*like 16S rRNA gene sequences were detected in about half of the lumbricid earthworms analyzed and in four other earthworm families (Davidson et al., 2013); since sequences from the same host families grouped together, the authors suggested host specificity of the *Flexibacter*-like symbionts. None of the other detected symbionts appeared to be widely present in earthworms (Davidson et al., 2013).

Based on their presence in multiple earthworm species, putative host specificity (Davidson et al., 2013), and vertical transmission (Davidson et al., 2010), we hypothesized that *Flexibacter*-like symbionts had a long evolutionary history with their earthworm hosts and with *Verminephrobacter*. The objectives of this study were (i) to investigate the putative host specificity of *Flexibacter*-like symbionts in more detail, and (ii) to elucidate the evolution of the tripartite symbiosis between earthworms, *Verminephrobacter*, and *Flexibacter*-like symbionts.

## Material and methods

### Collection and preparation of samples

A total of 18 earthworm species, 1–16 individuals of each (Supplementary Table S1), were collected in Denmark and Germany and identified by analytical keys (Sims and Gerard, 1985; Andersen, 1997). Specimens were cleaned with water, killed in 50% ethanol, and cut open along the dorsal line. Under a dissection microscope, the gut was carefully removed without disrupting it. For nucleic acid extraction, nephridia were collected by forceps from the anterior half of the gut-free worm and stored at −20°C. For fluorescence *in situ* hybridization (FISH), the gut-free body wall with attached nephridia was cut into smaller pieces (1–3 cm), fixed in 4% (w/v) paraformaldehyde for 2–3 h, washed twice in 1 × phosphate buffered saline (PBS), and stored in 70% ethanol/PBS at −20°C.

### Fluorescence *in situ* hybridization (FISH)

The localization of *Verminephrobacter* and *Flexibacter*-like bacteria in the nephridia was confirmed in two earthworm species; *Eisenia andrei* and *Allolobophora chlorotica*, using FISH. Hybridization was done at 35% formamide for 2.5 h directly on fixed pieces of body wall. The probes: LSB145-CY5 (Schweitzer et al., 2001), targeting some *Acidovorax* spp. and all *Verminephrobacter* spp., FLX226-CY3 (this study), targeting all *Flexibacter*-like symbionts, and EUB338,-II,-III-FAM (Daims et al., 1999), targeting all bacteria, were used in combination (Supplementary Table S1). The optimal formamide concentration of 35% for the probe FLX226 was determined using mathFISH (Yilmaz et al., 2011). The hybridizations were carried out according to published protocols (Pernthaler et al., 2001). Following hybridization and washing, the samples were counterstained with 4′,6-diamidino-2-phenylindole (DAPI; 1 μg mL^−1^) for 10 min on ice, rinsed thoroughly with water, and immersed in a 3:1 mixture of Vectashield (Vector Laboratories, Inc., Burlingame, CA) and Citifluor (Citifluor Ltd., London, UK). Single nephridia were dissected from the body wall and mounted on a glass slide prior to epifluorescence microscopy. Images were captured on an Axio Vert 200M epifluorescent light microscope, fitted with an Apotome for optical sectioning and an AxioCam MRm camera, all controlled by the AxioVision software (v4.8.1.0— all by Carl Zeiss, Jena, Germany).

### Nucleic acid extraction, PCR, cloning and sequencing

DNA was extracted from the dissected nephridia using the DNeasy Blood & Tissue kit (Qiagen, Valencia, CA, USA) following the protocol for animal tissue. DNA extracts were used for PCR amplification of both host and symbiont genes. All PCR reactions were run with the Taq master mix (VWR, Herlev, Denmark) containing 1.5 mM MgCl_2_, 0.2 mM of each dNTP, 0.1 units/μL VWR Taq polymerase, inert red dye, and 0.2 μM of each primer. Primers and thermal cycling protocols are summarized in Supplementary Table S2.

Two mitochondrially encoded genes, NADH dehydrogenase subunit II (ND2) and cytochrome c oxidase subunit I (COI), were targeted. These host genes are located adjacent to each other on the mitochondrial chromosome and were therefore amplified as one long fragment (ND2-COI) using the newly designed primers Lum-ND2-322F or Lum-ND2-370F, and Lum-COI-723R. Some COI sequences had been obtained in a previous study, in which cases only ND2 was amplified using the reverse primer Lum-COI-14R (Lund et al., 2010a, Supplementary Table S2). PCR fragments were purified (GenElute™ PCR Clean-Up kit, Sigma) and directly sequenced by Sanger sequencing (Macrogen, Korea) using the same primers as in the PCR reaction.

Earthworms were screened for the presence of *Flexibacter*-like symbiont 16S rRNA genes by specific PCR yielding a 174 bp-fragment (primers Flexi-145F and CF319aR, Supplementary Table S2). From the samples that tested positive, a longer fragment (primers Flexi-145F and 1492R, 1347 bp, Supplementary Table S2) was amplified, cloned, and sequenced. To obtain higher resolution in the phylogenetic trees, primers were designed for *rpoB* from *Verminephrobacter* (referred to as VrpoB; primers VrpoB-43F and VrpoB-1430R) and *Flexibacter*-like symbionts (referred to as FrpoB; primers FrpoB-1905F and FrpoB3213R, Supplementary Table S2). Primers were designed based on available *rpoB* sequences from the closest related organisms (*Flexibacter flexilis* for FrpoB and *V. eiseniae* EF2-01 and *V. aporrectodeae* At4 for VrpoB). *rpoB* was amplified, cloned using pGEM®-T easy vector system (Promega™) or TOPO® TA Cloning kit (Invitrogen™), and sequenced at GATC (Germany).

All obtained sequences have been submitted to GenBank under accession numbers KP420532-KP420720 and KM058238-KM058572 (Supplementary Table S1).

### Phylogenetic and statistical analyses

**Figure 2:**
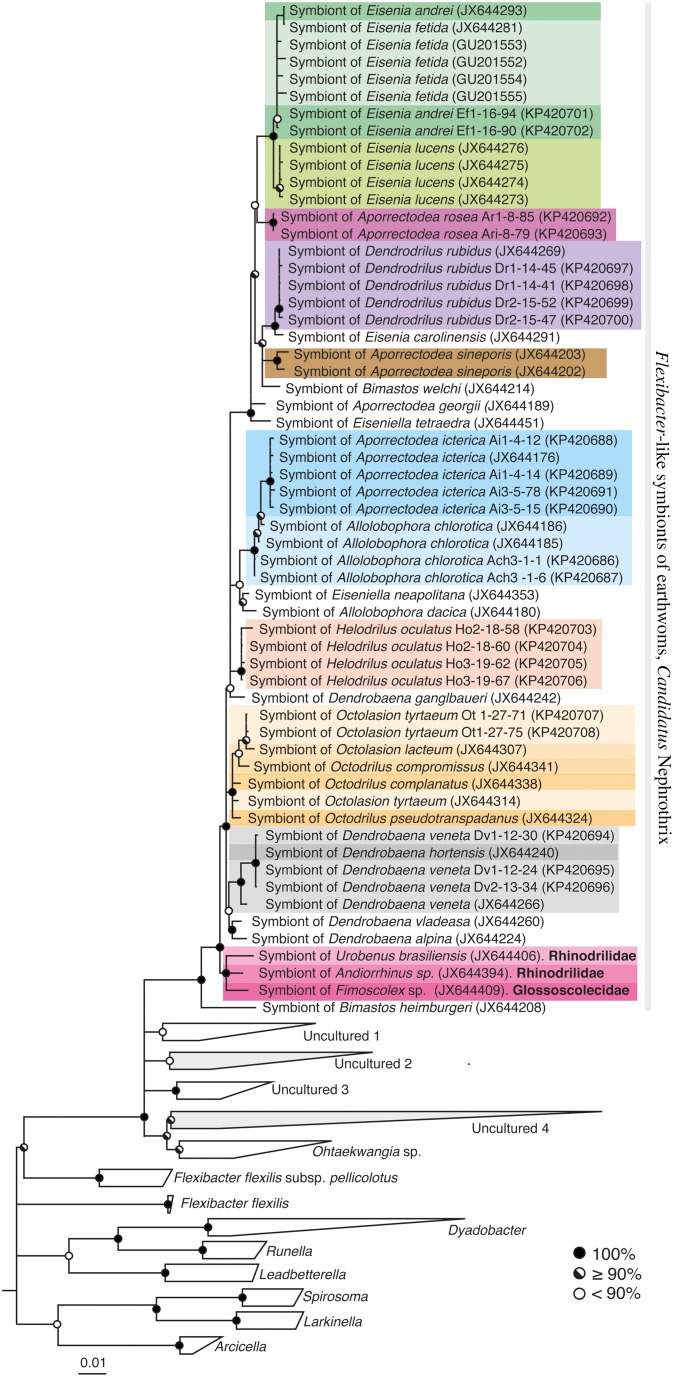
**16S rRNA gene sequence phylogeny of *Flexibacter*-like symbionts of earthworms and close relatives within the Cytophagaceae (Bacteroidetes)**. 60% majority rule consensus tree from Bayesian Inference. Circles on nodes show posterior probabilities according to legend. The *Flexibacter*-like symbionts of earthworms form a monophyletic group. If nothing else is indicated the earthworm species belongs to the family Lumbricidae. Gray clades contain other sequences detected in the nephridia of non-lumbricid earthworm families (Davidson et al., 2013): “Uncultured 2” contains three sequences from Megascolecidae species; *Arctiostrotus* sp. (JX644412), *Terisswalkerius covacevihae* (JX644411), *T. erici* (JX644410). “Uncultured 4” contains sequences from *Andiorrhinus* sp. (Pontoscolecidae, JX644395).

The newly obtained *Flexibacter*-like 16S rRNA gene sequences were aligned using the on-line SINA aligner (Pruesse et al., 2012) and imported into an existing ARB database (SSURef_111_SILVA_NR, (Ludwig et al., 2004; Pruesse et al., 2007). The ARB database was updated by identifying the closest relatives of the *Flexibacter*-like 16S rRNA gene sequences in GenBank using BLAST and importing them to the database if not already present. All published *Verminephrobacter* 16S rRNA gene sequences were also imported into the database. For constructing *Flexibacter*-like and *Verminephrobacter* 16S rRNA gene trees, a broad selection of related sequences from cultured organisms and environmental samples was included and the trees were calculated by Bayesian Inference using the software MrBayes (v3.2.3) (Ronquist et al., 2012). The substitution model was set to GTR + I + Γ and 60% majority rule consensus trees were calculated based on four simultaneous runs with six chains each and a sample frequency of 500. After 2,000,000 generations the potential scale reduction factors (PSRF) showed 1.000 indicating stationarity (Ronquist et al., 2012). The first 25% of the data was discarded as burn-in (Figure 2, Supplemental Figure S1).

**Figure 3:**
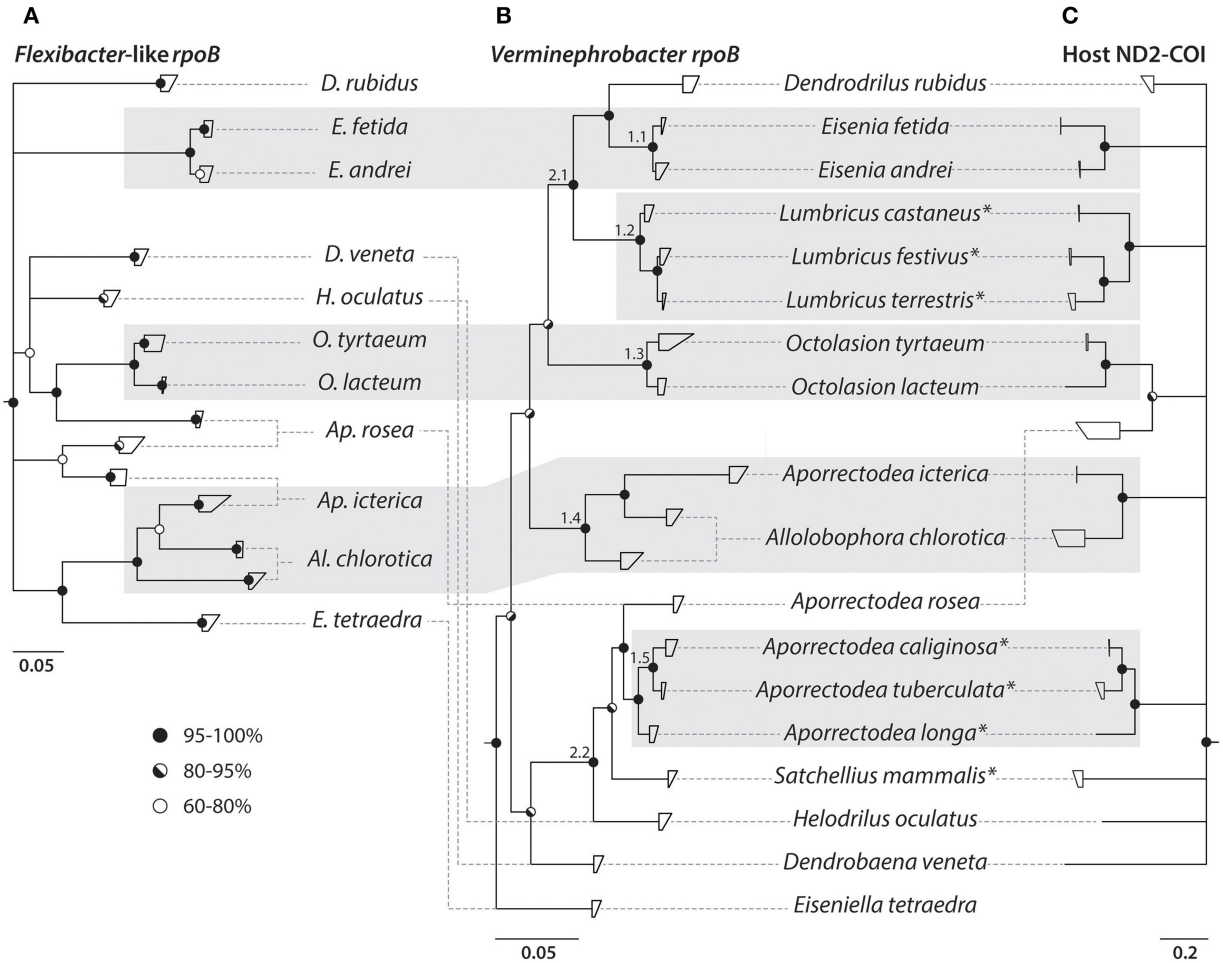
**Phylogenetic trees of earthworms and their *Verminephrobacter* and *Flexibacter*-like symbionts**. Nucleotide based *rpoB* phylogenies of **(A)** *Flexibacter*-like symbionts and **(B)** *Verminephrobacter*. **(C)** Earthworm phylogeny based on ND2-COI nucleotide sequences. Clades showing signs of co-diversification are highlighted by shaded boxes. All trees are majority rules (60%) consensus trees from Bayesian inference. Circles on nodes show posterior probabilities according to legend. Host species that do not harbor the *Flexibacter*-like symbiont are marked by an asterisk. Small numbers on branches in **(B)** are discussed in the text.

The host (ND2 and COI) and *rpoB* sequences were assembled and aligned in Geneious v. 5.6.7 (Biomatters LTD. Drummond et al., 2011) using the translation alignment algorithm for coding sequences. To determine an appropriate model of evolutionary nucleotide substitution for the individual target genes, the alignments where analyzed in jModelTest (Guindon and Gascuel, 2003; v2.1.5—Darriba et al., 2012). For each of the four target genes, the appropriate model was chosen based on the Akaike Information Criterion (AIC) (Akaike, 1974). The best fit substitution models were GTR + I + Γ for ND2, HKY + I + Γ for COI, GTR + Γ for VrpoB, and GTR + I + Γ for FrpoB. Phylogenetic trees were calculated by Bayesian Inference using MrBayes (v3.2.3) (Ronquist et al., 2012). The host genes (ND2 and COI) were concatenated and divided into six partitions; one for each codon position in both genes. The three partitions from each gene were set to evolve under the substitution models estimated with jModelTest. Both *rpoB* genes, VrpoB and FrpoB, were divided into three partitions (one for each codon position) and substitution models were chosen according to jModelTest. Majority rule consensus trees were calculated, based on two simultaneous runs with four chains each, and a sample frequency of 500. After 2,000,000 generations the PSRF showed 1.000 indicating stationarity (Ronquist et al., 2012). The first 500,000 generations were discarded as burn-in and the remainder were used to calculate 60% majority rule consensus trees (Figure 3).

A regular Mantel test was used to examine how well the host and symbiont phylogenies correspond to each other by testing the correlation between host (ND2-COI) and the corresponding symbiont rpoB distance matrices (host vs. FrpoB and host vs. VrpoB). For constructing the distance matrices highly similar sequences were grouped following the terminal nodes in Figure 3 and the between-group mean distance was calculated in MEGA6 (Tamura et al., 2013). Both Mantel tests were performed in R (R Core Team, 2013) as implemented in the package *vegan* (Oksanen et al., 2015) using the Pearson correlation method with 1000 permutations.

## Results

### Fluorescence in situ hybridization (FISH)

The presence of *Verminephrobacter* and *Flexibacter*-like bacteria was confirmed in *Al. chlorotica* and *E. andrei* (Figures 1B,C, respectively). In *Al*. *chlorotica* the *Flexibacter*-like symbionts line the lumen wall and *Verminephrobacter* is sparsely scattered throughout the lumen. The lumen is dominated by unidentified bacteria targeted by the general bacterial probe (EUB338,-II,-III). In *E. andrei* the *Verminephrobacter* lines the lumen wall, as typically found in other earthworm species (Davidson et al., 2010; Lund et al., 2014), and the *Flexibacter*-like symbionts fill up the lumen like cotton wool where individual cells are indistinguishable.

### Phylogeny of flexibacter-like earthworm symbionts

*Flexibacter*-like symbionts were detected in 11 of the 18 earthworm species investigated in this study; thus in total, 26 of the 51 lumbricid earthworm species ever analyzed scored positive for *Flexibacter*-like symbionts (Table 1). The 16S rRNA gene sequences of *Flexibacter*-like symbionts were also found in two sister groups to the Lumbricidae; one Glossoscolecidae (*Fimoscolex sp*.) and two Pontoscolecidae (*Andiorrhinus sp*. and *Urobenus brasiliensis*) (Davidson et al., 2013). The phylogeny of the *Flexibacter*-like symbionts was constructed using both 16S rRNA gene sequences (Figure 2) and *rpoB* sequences (Figure 3A). The 16S rRNA gene sequences were monophyletic within the Cytophagaceae and had low sequence similarities (90.6–92%) to the closest cultured relative; *Ohtaekwangia koreensis*. They were 91.5–94.2% similar to the closest environmental sequence identified by BLAST. The low intragroup genetic distance of only 4.8% makes the variation within the *Flexibacter*-like 16S rRNA gene sequences too low to reliably reconstruct the symbiont phylogeny.

**Table 1:** **Summary of earthworm species that have been investigated in this and previous studies for the presence/absence of V*erminephrobacter* and *Ca*. Nephrothrix**.

**Family**	**Species**	**Detection of V*erminephrobacter***		**Detection of *Ca*. Nephrothrix**		**Accession numbers^c^ for *Verminephrobacter* 16S rRNA gene sequences**	**Accession numbers^c^ for *Ca*. Nephrothrix 16S rRNA gene sequences**
		**Specific PCR LSB145F + 1492R**	**FISH with LSB145 probe**	**Specific PCR Flexi145F + CF319aR**	**FISH with FLX226 or Flexi145**		
Lumbricidae	*Allolobophora chlorotica*		+	+	+	3: FJ214195, FJ214204, JX644184	4: KP420686-87, JX644185-86
	*Allolobophora dacica*					1: JX644177	1: JX644180
	*Allolobophoridella eiseni*					1: JX644191	
	*Aporrectodea caliginosa*		+	**−**		7: FJ214188−89, FJ214202, JN809796−99	
	*Aporrectodea georgii*					1: JX644188	1: JX644189
	*Aporrectodea icterica*		+	+		3: FJ214196−97, JX644175	5: KP420688−91, JX644176
	*Aporrectodea longa*		+	**−**		2: FJ214190−1	
	*Aporrectodea meridionalis*					1: JX6442200	
	*Aporrectodea rosea*		+	+		2: FJ214194, FJ214203	2: KP420692−93
	*Aporrectodea sineporis*					1: JX644201	2: JX644202−03
	*Aporrectodea tuberculata*		+	**−**		9: AJ543437−38, FJ214186−87, FJ374774, JN809800−03	
	*Bimastos heimburgeri*					1: JX644207	1: JX644208
	*Bimastos welchi*						1: JX644214
	*Bimastos zeteki*					1: JX644218	
	*Dendrobaena alpina*						1: JX644224
	*Dendrobaena attemsi*		**−**				
	*Dendrobaena byblica*		**−**				
	*Dendrobaena clujensis*^d^						
	*Dendrobaena ganglbaueri*						1: JX644242
	*Dendrobaena hortensis*						1: JX644240
	*Dendrobaena lacustris*					1: JX644252	
	*Dendrobaena octaedra*	**−**	**−**	**−**			
	*Dendrobaena veneta*		+	+		3: FJ214198-99, JX644263	4: KP420694-95, KP420696, JX644266
	*Dendrobaena vladeasa*						1: JX644260
	*Dendrodrilus rubidus*			+		4: FJ214182-84, JX644267	5: KP420697-700, JX644269
	*Eisenia andrei*		+		+	3: FJ214179-80, JX644292	3: KP420701-02, JX644293
	*Eisenia balatonica*					2: JX644328-29	
	*Eisenia fetida*		+	+	+	17: AJ53439-40, DQ093612-13, DQ327662-69, GU201577-80, JX644280	1: JX644281
	*Eisenia lucens*					4: FJ214181, JX644270-72	4: JX644273-76
	*Eiseniella neapolitana*					1: JX644352	1: JX644353
	*Eiseniella tetraedra*					1: JX644350	1: JX644351
	*Eisenoides carolinensis*					2: FJ214185, JX644290	1: JX644291
	*Healyella jordanis*^d^						
	*Helodrilus oculatus*		+	+		2: FJ214192-93	4: KP420703-06
	*Lumbricus castaneus*		+	**−**		3: FJ214175-76, JX644301	
	*Lumbricus centralis*					1: JX644299	
	*Lumbricus festivus*		+	**−**		3: FJ214170, FJ214173-74	
	*Lumbricus friendi*					1: JX644333	
	*Lumbricus polyphemus*					1: JX644336	
	*Lumbricus rubellus*		+	**−**		5: AY154496, FJ214177-78, JX644334-35	
	*Lumbricus terrestris*		+	**−**		5: FJ214171-72, AJ543435-36, JX644296	
	*Octodrilus complanatus*					1: JX644337	1: JX644338
	*Octodrilus compromissus*					1: JX644339	1: JX644341
	*Octodrilus gradinescui*					1: JX644343	
	*Octodrilus permagnus*					2: JX644319-20	
	*Octodrilus pseudotranspandanum*						1: JX644324
	*Octolasion cyaneum*					2: FJ214208, JX644305	
	*Octolasion lacteum*					2: FJ214207, JX644306	1: JX644307
	*Octolasion sp*.					2: FJ214201, JX644309	
	*Octolasion tyrtaeum*			+		4: FJ214200, FJ214205-06, JX644311	3: KP420707-08, JX644314
	*Satchellius mammalis*			**−**		*rpoB*^a^	
Glossoscolecidae	*Fimoscolex* sp.						JX644208
Pontoscolecidae^b^	*Andiorrhinus* sp.						JX644394
	*Urobenus brasiliensis*						JX644406

*For a complete sample list for this study see Supplementary Table S1. Positive test “+”. Negative test “−,” If nothing is indicated the test has not been performed. Accession numbers are given for all published Verminephrobacter and Ca. Nephrothrix 16S rRNA gene sequences obtained from clone libraries^c^*.

a *^a^Verminephrobacter was detected in a rpoB gene clone library*.

b *This family is called Rhinodrillidae in Davidson et al. (2013) but was reclassified as a Pontoscolecidae in James and Davidson (2012)*.

c *References for accession numbers: [AJ… … ] Schramm et al. (2003) [DQ32….] Pinel et al. (2008) [DQ09….] Davidson et al. (2010) [FJ……] Lund et al. (2010a) [JN……] Lund et al. (2012); [JX……] Davidson et al. (2013) [KP……] This study*.

d *General 16S rRNA clone libraries showed no Verminephrobacter *or* Ca. *Nephrothrix*, no attempts of specific detection have been made*.

**Figure 4:**
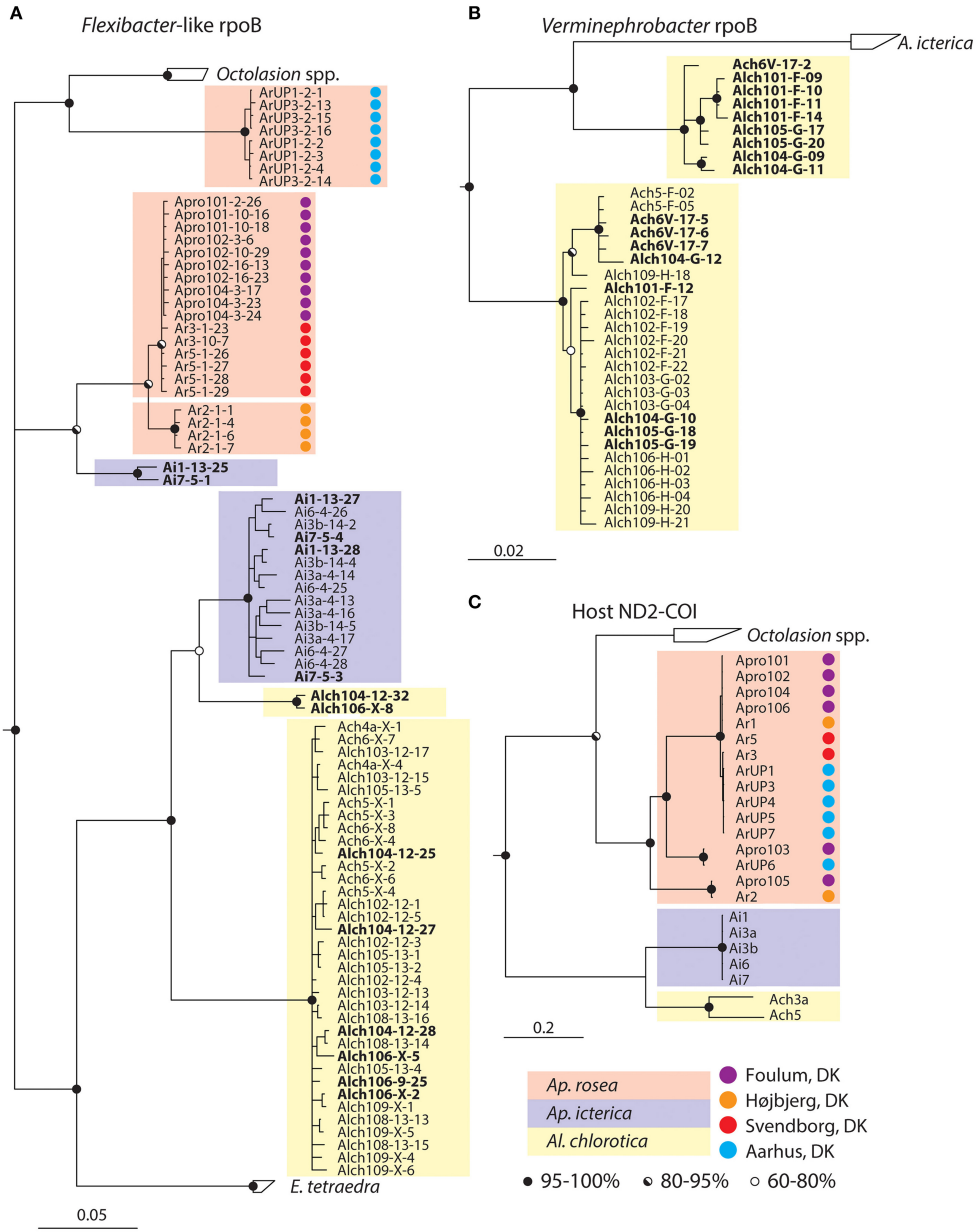
**Details of selected clades from trees in Figure 3. (A)** *Flexibacter*-like symbionts, **(B)** *Verminephrobacter*, and **(C)** earthworms. Different earthworm species are highlighted with background-colored boxes according to legend. Names in bold highlight clones from the same individual host that are associated with two different clades. The first part of the clone name indicates host individual. Background-Colored circles denote the geographic origin of host individuals according to legend. All trees are majority rule (60%) consensus trees from Bayesian inference. Circles on nodes are posterior probabilities according to legend.

The *rpoB*-based tree has low resolution of the basal branches but the *Flexibacter*-like symbionts from different host species can clearly be distinguished and are indeed species-specific (Figure 3A). However, *Aporrectodea rosea, Al. chlorotica*, and *Ap. icterica* each carry two distinct symbiont types that are not monophyletic with each other (Figures 3A, 4A). The *Flexibacter*-like symbionts of *Ap. rosea* form one group clustering with *Octolasion* and the other with *Ap. icterica*. For the *Ap. icterica* symbionts, one group clusters with *Ap. rosea* and the other with *Al. chlorotica*. In *Al. chlorotica* both groups cluster with *Ap. icterica*, but together they are not monophyletic. In both *Ap. icterica* and *Al. chlorotica* the two variants of *Flexibacter-*like symbionts coexist in the same individual worms (Figure 4A); this was found in two individuals of each species. For *Ap. rosea*, however, all of the sequences from one of the symbiont clusters originate from worms of the same geographic origin (Figure 4A).

### Phylogeny of verminephrobacter symbionts

Two separate *Verminephrobacter* phylogenies were constructed using *rpoB* (Figure 3B) and 16S rRNA gene sequences (Supplementary Figure S1). All 16S rRNA gene sequences were retrieved from GenBank. The *rpoB*-based phylogeny has a better resolution of closely related strains, like *Eisenia sp*. and *Lumbricus* sp., which cannot be resolved using 16S rRNA gene sequences (Supplementary Figure S1). However, the *rpoB*-based tree has low posterior probabilities (down to 80%) toward the basal nodes of the tree (Figure 3B). There are large discrepancies in the basal branching pattern between the two phylogenies, but the terminal clades labeled 1.1–1.5 are found in both trees. The nodes labeled 2.1 and 2.2 are also found in both trees but the branching patterns within these two clades differ in the two phylogenies.

### Comparison of host and symbiont phylogenies

The earthworm phylogeny is based on concatenated ND2 and COI sequences (Figure 3C). Even though the combined PCR fragment is about 1400 bp, only 921 bp of high quality sequence could be retrieved after sequencing. Five clusters could be resolved with high (94–100%) posterior probabilities but the basal branching of the tree was not resolved, which is a common problem in earthworm phylogenies (reviewed by Chang and James, 2011). It was not possible to amplify ND2-COI from *Eiseniella tetraedra*.

All of the resolved groups in the earthworm phylogeny are reflected in the *Verminephrobacter rpoB* and 16S rRNA gene trees, with *A. rosea* as an obvious exception (Figures 3B,C, Supplementary Figure S1). As for the *Flexibacter*-like symbionts, the *Verminephrobacter* symbionts in *Al. chlorotica* formed two distinct, coexisting clades, one of which grouped with the symbionts of *Ap. icterica* (Figures 3B, 4B). The Mantel test showed a strong positive relationship (*r* = 0.5065; *P* < 0.001) between host and *Verminephrobacter* genetic distances based on ND2-COI and *rpoB*, respectively.

Due to low resolution of both the host and the *rpoB*-based tree of *Flexibacter*-like symbionts it is not possible to evaluate the degree of congruency between the two (Figures 3A,C). However, for closely related earthworm species there are signs of co-speciation, i.e., *Eisenia fetida* and *E. andrei* (and *E. lucens*, Figure 1), *Octolasion tyrtaeum* and *O. lacteum*. Also, leaving aside the second symbiont clades (see above), the *Flexibacter*-like symbionts, *Verminephrobacter*, and the hosts, all have the same clustering of *Al. chlorotica* and *Ap. icterica* (Figure 3). These signs of co-speciation are also reflected in the Mantel test which showed a moderate positive relationship (*r* = 0.3753; *P* < 0.01) between host and *Flexibacter*-like genetic distances based on ND2-COI and *rpoB*, respectively. Meanwhile, there are clear signs of host switching in the three worm species all having two distinct *Flexibacter*-like symbiont groups (*Ap. rosea, Ap. icterica*, and *Al. chlorotica*, Figure 3A).

When comparing the *rpoB*-based phylogenies of *Verminephrobacter* and the *Flexibacter*-like symbionts (Figures 3A,B) there are clear signs of incongruences in the deeper branching patterns.

## Discussion

### Flexibacter-like symbiont phylogeny and proposal of the candidate genus nephrothrix

The *Flexibacter*-like symbionts of lumbricid earthworms clearly form their own monophyletic cluster based both on 16S rRNA (Figure 2) and *rpoB* (Figure 3A) gene sequences. The monophyletic group is very coherent and distantly related to other known organisms; based on 16S rRNA gene sequences they have an intragroup genetic distance of only 4.8% and a low similarity (91.5–94.2%) to other known sequences. *Flexibacter*-like symbionts are widespread in the Lumbricidae, where they are present in about 50% of the investigated worms (Table 1; Davidson et al., 2013); absence was confirmed in eight species using specific PCR (Table 1). The *Flexibacter*-like symbionts were also found in one member of the Glossoscolecidae and two of Pontoscolecidae (Table 1, Davidson et al., 2013), both sister families to Lumbricidae (James and Davidson, 2012). These *Flexibacter*-like symbionts of non-lumbricid worms form their own monophyletic clade toward the base of the group (the most basal sequence is from *Bimastos heimburgeri*, Lumbricidae, Figure 2). The presence of *Flexibacter*-like symbionts in the nephridia of these worms has not been confirmed with FISH. Davidson et al. (2013) also reported the presence of *Flexibacter*-like bacteria in other earthworm families; however, their 16S rRNA gene sequences group outside the above described monophyletic cluster (Figure 2). Apparently, they represent different evolutionary lineages and are therefore not considered part of the novel symbiont genus proposed below (Figure 2).

For the monophyletic *Flexibacter*-like symbionts of lumbricid earthworms, the low similarity (91.5–94.2%) to other known 16S rRNA gene sequences, combined with their unique habitat in earthworm nephridia and their conspicuous morphology, supports the establishment of a novel genus. In spite of numerous attempts, the symbionts could not be cultivated yet (Marie Lund, unpublished; Flávia Viana, personal communication); they can, however, be easily identified *in situ* by the newly developed FISH probe (FLX226, Supplementary Table S2). In accordance with the Report of the ad hoc committee for the re-evaluation of the species definition in bacteriology (Stackebrandt et al., 2002), we therefore propose the establishment of a candidate genus and the name “*Candidatus* Nephrothrix” [Ne.phro.thrix. Gr. n. *nephros*, kidney; Gr. fem. n. *thrix*, hair; N. L. fem. n. *Candidatus* Nephrothrix, kidney (associated) hair], which reflects the specific habitat (earthworm nephridia) and the slender, hair-like morphology of these symbionts. As a first representative of the candidate genus, we suggest the specific symbiont of *Eisenia fetida* to be named “*Ca*. N. davidsonii” in honor of Seana Davidson, the scientist who first discovered the *Flexibacter*-like symbionts in earthworms.

### Ca. Nephrothrix switches earthworm host

It can not be concluded if *Ca*. Nephrothrix is an ancient co-evolving symbiont of earthworms due to the poor basal resolution of both phylogenies (Figures 3A,C). There are some indications of co-evolution (Figures 3A,C), which is supported by the Mantel test showing a moderate positive correlation between the *Ca*. Nephrothrix and host distance matrices. However, there are also clear examples of host switching where distinct *Ca*. Nephrothrix types are found in the same host species (Figure 3A). Also, the monophyletic group of non-lumbricid derived 16S rRNA gene sequences toward the base of the *Ca*. Nephrothrix cluster (Figure 2) indicates a host switch because the most basal sequence in the group is from a lumbricid worm. Whether the first host of *Ca*. Nephrothrix was a lumbricid or belonged to one of the sister families is unknown but given the basal grouping of the sequences from Glossoscolecidae and Pontoscolecidae it is possible that *Ca*. Nephrothrix originated in a non-lumbricid worm.

After each host switch, *Ca*. Nephrothrix apparently became species-specific, and during two more recent, separate host speciation events (in *Eisenia* and in *Octolasion*) *Ca*. Nephrothrix possibly co-diversified with its host (Figure 3A, Supplementary Figure S2); in which case *Ca*. Nephrothrix was already present in the ancestors of *Eisenia* and *Octolasion*. Alternatively, *Eisenia* sp. and *Octolasion* sp. speciated before colonization and picked up closely related *Ca*. Nephrothrix at a later point. This would result in the same pattern of apparent co-speciation.

Interestingly, in three earthworm species (*Ap. rosea, Ap. icterica*, and *Al. chlorotica*) two distinct *Ca*. Nephrothrix *rpoB* variants are found (Figure 4A). In *Ap. rosea*, all of the sequences in one cluster originate from worms collected at the same site, thus possibly representing a local host-switching event. In *Ap. icterica* and *Al. chlorotica*, on the other hand, the two *rpoB* variants coexist in the same worm individuals. The *rpoB* gene is generally found to be a single copy gene (Case et al., 2007), and we therefore assume that the gene variants represent two different *Ca*. Nephrothrix populations. In both cases of co-existing *Ca*. Nephrothrix populations, the symbionts from one of the populations are much more common than from the other (Figure 4A). When considering the tree topology, it is likely that the dominant populations represent the original *Ca*. Nephrothrix symbionts, which have co-diversified with their hosts during the speciation event between *Ap. icterica* and *Al. chlorotica*. The secondary *Ca*. Nephrothrix populations may then originate from recent host-switching events.

The co-existing symbiont populations in the same earthworm species or individuals show that *Ca*. Nephrothrix can switch between earthworm hosts. After each host-switching event, they became species-specific again, indicating that they adapted to the new host environment. Host switching can only occur if *Ca*. Nephrothrix is sometimes horizontally transmitted, thus the vertical transmission previously demonstrated in *E. fetida* (Davidson et al., 2010) must be leaky in at least some earthworm species. It is not known if the greatest barrier for host switching is (i) lack of opportunity, i.e., worms rarely encounter non-native symbionts, or (ii) species-specificity where non-native symbionts are excluded because they do not respond correctly to host signals. It is furthermore unknown if the horizontal transmission occurs by direct contact between the different worm species or if there is an environmental reservoir of *Ca*. Nephrothrix.

### Distribution of verminephrobacter and co-speciation with its earthworm host

*Verminephrobacter* are widespread in the Lumbricid earthworms, where they are present in about 80% of the investigated species (Table 1, Lund et al., 2010a; Davidson et al., 2013). However, most investigations have been based on bacterial 16S rRNA gene clone libraries rather than *Verminephrobacter*–specific FISH or PCR assays; thus if the symbionts were not detected it could mean that they were overlooked rather than truly absent. The absence of *Verminephrobacter* has only been confirmed by specific FISH in three worm species; *Dendrobaena octaedra, D. attemsi*, and *D. byblica* (Table 1, Lund et al., 2010a; Davidson et al., 2013). Since *Verminephrobacter* was generally not detected in *Dendrobaena* species, it is likely indeed absent from this genus.

Davidson et al. (2013) also reported *Verminephrobacter* 16S rRNA gene sequences in two worms from the earthworm family Microchaetidae. These two sequences were presented in a tree with one large unresolved group of *Acidovorax* and *Verminephrobacter*. In contrast, our analyses clearly established *Verminephrobacter* from Lumbricidae as monophyletic genus with high confidence (Supplementary Figure S1), and thus confirmed earlier phylogenies (Schramm et al., 2003; Pinel et al., 2008; Lund et al., 2010a). In addition, the two sequences from Microchaetidae clustered with the genus *Acidovorax* and therefore do not belong within *Verminephrobacter*.

The poor basal resolution of both host and *Verminephrobacter* trees (Figures 3B,C, Supplemental Figure S1) prevents a direct proof of co-diversification of the ancient symbiotic partners. However, clear congruence of the five clades that could be resolved in both host and *Verminephrobacter* trees does support the hypothesis of co-evolution along with the Mantel test showing a strong positive relationship between the host and *Verminephrobacter* distance matrices. Meanwhile, the *Ap. rosea* symbiont is a clear example of host switching.

Another putative example of host switching between closely related worms is *Verminephrobacter* in *Al. chlorotica*: Interestingly, four *Al. chlorotica* individuals from two geographic locations (Figure 4C, Supplementary Table S1) have two coexisting *rpoB* gene variants. These gene variants are likely to originate from two distinct *Verminephrobacter* populations since *rpoB* is generally a single copy gene (Case et al., 2007). One of the *rpoB* variants groups with *Ap. icterica* and could therefore have originated from this species. This is the only reported example of distinct *Verminephrobacter* types coexisting in the same host. FISH analysis shows that *Verminephrobacter* only comprises a small fraction of the nephridial community in *Al. chlorotica* (Figure 1B, (Lund et al., 2010a)) and that *Verminephrobacter* is located in the lumen, rather than being associated with the ampulla wall as usual (Figures 1B,C, Davidson et al., 2010; Lund et al., 2014). Potentially, *Al. chlorotica* is in the process of losing its *Verminephrobacter* symbiont; signals involved in symbiont specificity may already be weakened, thus allowing the colonization by a non-native *Verminephrobacter*.

### An ancient and a young symbiont

The age of the two symbiont groups can be estimated by comparison with the age of the earthworm hosts. In a recently published Lumbricidae phylogeny, the family was estimated to originate during the Cretaceous about 110 MYA (95% confidence interval; 100–120 MYA) and the split to the most basal worm species with *Verminephrobacter* (*Allolobophora dacia*) happened 95 MYA (95% confidence interval 90–100 MYA) (Domínguez et al., 2015). Thus, *Verminephrobacter* has likely been associated with lumbricid earthworms for about 100 MY. Since *Ca*. Nephrothrix has apparently switched hosts it is not possible to date the origin of this symbiosis. However, the putative recent co-speciation events with *Octolasion* sp. and *Eisenia* sp. facilitate a minimum estimate of the age of the group. According to Domínguez et al. (2015), *O. tyrtaeum* and *O. lacteum* diverged during the Paleogene about 40 million years ago (MYA, 95% confidence interval; 15–70 MYA), while *Eisenia lucens* diverged from *E. fetida/E. andrei* about 45 MYA (95% confidence interval; 35–55 MYA). Thus, *Ca*. Nephrothrix has likely been associated with the Lumbricidae for a minimum of 45 MY. Furthermore, the intragroup genetic distance in the 16S rRNA gene in *Verminephrobacter* (7.3%) is higher than in *Ca*. Nephrothrix (4.8%), supporting that the genus *Verminephrobacter* is older than *Ca*. Nephrothrix.

## Conclusion and perspective

In the tripartite symbiosis between earthworms*, Verminephrobacter* and *Ca*. Nephrothrix, the two bacterial partners have followed markedly different evolutionary trajectories. Symbionts from the novel candidate genus, *Ca*. Nephrothrix, are found in approximately half of all lumbricid earthworm species, they have apparently switched hosts, and subsequently adapted and become species-specific. In contrast, *Verminephrobacter* show signs of long-term cospeciation with their host, they are present in almost all lumbricid earthworms, and have rare host switching events. *Verminephrobacter* is thus likely to have originated in the last common ancestor of lumbricid earthworms 100 MYA (Lund et al., 2010a; Domínguez et al., 2015, whereas *Ca*. Nephrothrix is much younger with an estimated age of ≥45 MY. This scenario still allows for co-occurrence of the two symbionts in a range of earthworm species for at least 45 MY, and thus plenty of opportunity for interspecies genetic exchange as predicted by Kjeldsen et al. (2012) to explain *Verminephrobacter* genome evolution. In support of this possibility, natural competence and transformation of *Verminephrobacter* within earthworm cocoons has recently been demonstrated (Davidson et al., 2014). Whether *Ca*. Nephrothrix has left its genetic trace in *Verminephrobacter* and *vice versa* remains to be shown.

### Conflict of interest statement

The authors declare that the research was conducted in the absence of any commercial or financial relationships that could be construed as a potential conflict of interest.
