# Supplementary material for: Evolution of the tripartite symbiosis between earthworms, Verminephrobacter and Flexibacter-like bacteria
Source: Front Microbiol. 2015 May 27;6:529. doi: 10.3389/fmicb.2015.00529 (PMC4445045; doi:10.3389/fmicb.2015.00529)
Supplement: Supplementary file 1 [file Table1.PDF]

Table S1: Species list, sampling location, and genes sequenced. Shaded areas indicate earthworm species lacking *Flexibacter*-like symbionts. GenBank accession numbers are given for all sequences in the analysis.

			**Earthworms**		***Verminephrobacter***		***Flexibacter*-like**	
**Earthworm species**	**Sample**	**Location**	**ND2**	**COI**	***rpoB***	**16S**	***rpoB***	**16S**
Allolobophora chlorotica	Ach3a	Svendborg, DK	KP420609	FJ214210	-	-	-	2: KP420686-87
(Savigny)	Ach4a	Svendborg, DK	-	-	-	-	2: KM058238-39	-
	Ach5	Elsted, Aarhus, DK	KP420610	FJ214231	2: KM058383-84	1: FJ214204	4: KM058240-43	-
	Ach6	Elsted, Aarhus, DK	-	-	4: KM058385-88	-	4: KM058244-48	-
	Alch101	Nienstädt, DE	-	-	5: KM058389-93	-	-	-
	Alch102	Nienstädt, DE	-	-	6: KM058394-99	-	4: KM058248-51	-
	Alch103	Nienstädt, DE	-	-	3: KM058400-02	-	4: KM058252-55	-
	Alch104	Nienstädt, DE	-	-	4: KM058403-06	-	4: KM058256-59	-
	Alch105	Nienstädt, DE	-	-	4: KM058407-10	-	4: KM058260-63	-
	Alch106	Nienstädt, DE	-	-	4: KM058411-14	-	4: KM058264-67	-
	Alch108	Nienstädt, DE	-	-	-	-	4: KM058268-71	-
	Alch109	Nienstädt, DE	-	-	3: KM058415-17	-	4: KM058272-75	-
Aporrectodea caliginosa	Acal107	Nienstädt, DE	KP420606	KP420532	2: KM058418-19	-		
(Savigny)	Acc1	Svendborg, DK	KP420607	KP420533	-	-		
	Acc2	Svendborg, DK	-	-	2: FJ214281-82	1: FJ214188		
	Acc3	Svendborg, DK	KP420608	KP420534	-	1: FJ214189		
Aporrectodea icterica	Ai1	Højbjerg, DK	KP420611	KP420535	5: KM058420-24	1: FJ214197	3: KM058276-78	2: KP420688-89
(Savigny)	Ai3a	Højbjerg, DK	KP420612	KP420536	2: KM058425-26	1: FJ214196	4: KM058279-82	2: KP420690-91
	Ai3b	Højbjerg, DK	KP420613	KP420537	4: KM058427-30	-	3: KM058283-85	-
	Ai6	Højbjerg, DK	KP420614	KP420538	4: KM058431-34	-	4: KM058286-89	-
	Ai7	Højbjerg, DK	KP420615	KP420539	-	-	3: KM058290-92	-
Aporrectodea longa	Al3	Aarhus, DK	KP420616	FJ214222	2: FJ214284-85	1: FJ214190		
(Ude)	Al4	Aarhus, DK	-	-	2: FJ214286-87	1: FJ214191		
	Al7	Aarhus, DK	-	-	2: FJ892716-17	-		
Aporrectodea rosea	Ar1	Højbjerg, DK	KP420623	KP420546	5: KM058435-38, FJ214288	1: FJ214194	-	2: KP420692-93
(Savigny)	Ar2	Højbjerg, DK	KP420624	KP420547	-	-	4: KM058293-96	-
	Ar3	Svendborg, DK	KP420625	KP420548	5: KM058439-43	-	2: KM058297-98	-
	Ar5	Svendborg, DK	KP420626	KP420549	5: KM058444-47, FJ214308	1: FJ214203	4: KM058299-302	-
	ArUP1	Aarhus, DK	KP420627	KP420550	4: KM058448-51	-	4: KM058303-06	-
	ArUP3	Aarhus, DK	KP420628	KP420551	1: KM058452	-	4: KM058307-10	-
	ArUP4	Aarhus, DK	KP420629	KP420552	-	-	-	-
	ArUP5	Aarhus, DK	KP420630	KP420553	-	-	-	-
	ArUP6	Aarhus, DK	KP420631	KP420554	-	-	-	-
	ArUP7	Aarhus, DK	KP420632	KP420555	-	-	-	-
	Apro101	Foulum, DK	KP420617	KP420540	3: KM058453-55	-	3: KM058311-13	-
	Apro102	Foulum, DK	KP420618	KP420541	4: KM058456-59	-	4: KM058314-17	-

Table S1 (*continued*): Species list, sampling location, and genes sequenced. Shaded areas indicate earthworm species lacking *Flexibacter*-like symbionts. GenBank accession numbers are given for all sequences in the analysis.

(continued - 2 of 3)			Earthworms		*Verminephrobacter*		*Flexibacter*-like	
**Earthworm species**	**Sample**	**Location**	**ND2**	**COI**	***rpoB***	**16S**	***rpoB***	**16S**
Aporrectodea rosea	Apro103	Foulum, DK	KP420619	KP420542	4: KM058460-64	-		-
(Savigny)	Apro104	Foulum, DK	KP420620	KP420543	3: KM058465-66	-	3: KM058318-20	-
	Apro105	Foulum, DK	KP420621	KP420544	-	-	-	-
	Apro106	Foulum, DK	KP420622	KP420545	3: KM058467-69	-	-	-
Aporrectodea tuberculata	At1	Højbjerg, DK	KP420633	KP420556	-	-		
(Eisen)	At2	Aarhus, DK	KP420634	FJ214223	2: FJ214289-90	1: FJ214174		
	AtG1	Bayreuth, Germany	KP420635	KP420557	-	-		
	AtG2	Bayreuth, Germany	KP420636	KP420558	-	-		
	AtG3	Bayreuth, Germany	KP420637	KP420559	-	-		
	AtG4	Bayreuth, Germany	KP420638	KP420560	-	-		
	AtM4	Mårslet, DK	KP420639	KP420561	-	-		
	AtM5	Mårslet, DK	KP420640	KP420562	-	-		
	AtM6	Mårslet, DK	KP420641	KP420563	-	-		
	At4culture	unknown	-	-	1: FJ214311	-		
Dendrodrilus rubidus	Dr1	Højbjerg, DK	KP420642	KP420564	4: KM058473-74, FJ214295-96	1: FJ214182	4: KM058332-35	2: KP420697-98
(Savigny)	Dr2	Højbjerg, DK	KP420644	KP420566	7: KM058475-79, FJ214309, FJ214297	1: FJ214184	-	2: KP420699-700
	Dr10	Konnevesi, FI	KP420643	KP420565	5: KM058480-84	1: FJ214183	3: KM058336-38	-
Dendrobaena veneta	Dv1	Netherlands (VC)	-	-	1: FJ214292	1: FJ214198	3: KM058321-23	2: KP420694-95
(Bouché)	Dv2	Netherlands (VC)	-	-	2: FJ214293-94	1: FJ214199	4: KM058324-27	1: KP420696
	Dv3	Netherlands (VC)	KP420645	KP420567	3: KM058470-72	-	4: KM058328-31	-
Eisenia andrei	EaCA10	Aarhus, DK	KP420646	KP420568	-	-	-	-
(Bouché)	EaCA11	Aarhus, DK	KP420647	KP420569	-	-	-	-
	EaCA12	Aarhus, DK	KP420648	KP420570	-	-	-	-
	EaCA13	Aarhus, DK	KP420649	KP420571	-	-	-	-
	EaM2	Mårslet, DK	KP420654	KP420576	-	-	-	-
	EaM3	Mårslet, DK	KP420655	KP420577	-	-	-	-
	EaM4	Mårslet, DK	KP420656	KP420578	-	-	-	-
	EaM10	Mårslet, DK	KP420650	KP420572	8: KM058485-92	-	-	-
	EaM11	Mårslet, DK	KP420651	KP420573	4: KM058493-96	-	-	-
	EaM12	Mårslet, DK	KP420652	KP420574	4: KM058497-00	-	-	-
	EaM13	Mårslet, DK	KP420653	KP420575	3: KM058501-03	-	-	-
	Ef1	Svendborg, DK	KP420657	KP420579	6: KM058504-07, FJ214298-99	1: FJ214179	-	KP420701-02
	Ef2	Svendborg, DK	KP420658	KP420580	6: KM058508-11, FJ214273, FJ214300	1: FJ214180	-	-
	Ef3b	Svendborg, DK	KP420659	KP420581	4: KM058512-15	-	4: KM058339-42	-

Table S1 (*continued*): Species list, sampling location, and genes sequenced. Shaded areas indicate earthworm species lacking *Flexibacter*-like symbionts. GenBank accession numbers are given for all sequences in the analysis.

(continued – 3 of 3)			**Earthworms**		***Verminephrobacter***		***Flexibacter*-like**	
**Earthworm species**	**Sample**	**Location**	**ND2**	**COI**	***rpoB***	**16S**	***rpoB***	**16S**
Eisenia fetida	EfG1	Bayreuth, Germany	KP420660	KP420582	-	-	4: KP420709-12	-
(Savigny)	EfG2	Bayreuth, Germany	KP420661	KP420583	6: KM058516-21	-	4: KP420713-16	-
	EfG3	Bayreuth, Germany	KP420662	KP420584	5: KM058522-26	-	4: KP420717-20	-
	EfG4	Bayreuth, Germany	KP420663	KP420585	-	-	-	-
Eiseniella tetraedra	Et1	Moesgaard, DK	-	-	4: KM058527-30	-	4: KM058343-46	-
(Savigny)	Et2	Moesgaard, DK	-	-	-	-	4: KM058347-50	-
	Et3	Moesgaard, DK	-	-	4: KM058531-34	-	4: KM058351-54	-
Helodrilus oculatus	Ho2	Moesgaard, DK	-	-	1: FJ214303	1: FJ214192	4: KM058355-58	2: KP420703-04
(Hoffmeister)	Ho3	Moesgaard, DK	KP420664	KP420586	2: FJ214304, FJ214310	1: FJ214193	4: KM058359-62	2: KP420705-06
Lumbricus castaneus	Lc1	Svendborg, DK	-	-	2: FJ214274-75	1: FJ214175		
(Savigny)	Lc2	Svendborg, DK	-	-	2: FJ214276, FJ214301	1: FJ214176		
	Lc21	Aarhus, DK	KP420665	KP420587	1: FJ892723	-		
	Lc22	Aarhus, DK	KP420666	KP420588	1: FJ892722	-		
	Lc23	Aarhus, DK	KP420670	KP420592	-	-		
	Lc24	Moesgaard, DK	KP420671	KP420593	-	-		
Lumbricus festivus	Lf1	Aarhus, DK	KP420667	KP420589	5: KM058535-39	1: FJ214170		
(Savigny)	Lf2	Svendborg, DK	KP420668	KP420590	2: FJ892720-21	1: FJ214174		
	Lf3	Svendborg, DK	-	-	2: FJ214277, FJ214307	1: FJ214173		
	Lfr	Holme, Aarhus, DK	KP420669	KP420591		-		
Lumbricus terrestris	Lt1	Aarhus, DK	KP420672	FJ214212	-	-		
(Linnaeus)	Lt2	Aarhus, DK	KP420673	FJ214211	-	1: FJ214172		
	Lt6	Aarhus, DK	-	-	2: FJ214278, FJ214291	-		
	Lter	unknown	NC001673	NC001673	-	-		
Octolasion lacteum (Örley)	Ol4	Højbjerg, DK	KP420676	KP420596	4: KM058540-43	-	4: KM058363-66	-
Octolasion tyrtaeum	Ot1	Højbjerg, DK	KP420674	KP420594	2: FJ214305-06	1: FJ214200	4: KM058367-70	2: KP420707-08
(Savigny)	Ot3	Højbjerg, DK	KP420675	KP420595	4: KM058544	1: FJ214205	4: KM058371-74	-
	Ot6	Bayreuth, DE	KP420677	KP420597	4: KM058548	1: FJ214206	4: KM058375-78	-
	Ot7	Bayreuth, DE	-	-	3: KM058552	-	4: KM058379-82	-
Satchellius mammalis	Sm1	Holme, Aarhus, DK	2: KP420678-79	2: KP420598-99	5: KM058555-59	-		
(Savigny)	Sm2	Holme, Aarhus, DK	2: KP420680-81	2: KP420600-01	4: KM058560-63	-		
	Sm3	Holme, Aarhus, DK	2: KP420682-83	2: KP420602-03	4: KM058564-67	-		
	Sm4	Holme, Aarhus, DK	2: KP420684-85	2: KP420604-05	5: KM058568-72	-		
