# Supplementary material for: Evolution of the tripartite symbiosis between earthworms, Verminephrobacter and Flexibacter-like bacteria
Source: Front Microbiol. 2015 May 27;6:529. doi: 10.3389/fmicb.2015.00529 (PMC4445045; doi:10.3389/fmicb.2015.00529)
Supplement: Supplementary file 2 [file Table2.PDF]

Table S2: PCR primers and FISH probes. Thermal cycling conditions are given for all primer pairs.

	Target gene	Primer/probe	Sequence (5'-3')	Thermal cycling			Cycles	Reference
PCR primers	Earthworm	Lum-ND2-322F or	TGT CAT YTC TGA TAC CCR TCA GT	Initial denaturation	94˚C	03:00		This study
	ND2-COI	370F	TGC CTD ATY YTA TCN WCM TG	Denaturation	94˚C	00:30		
		Lum-COI-723R	TAK ACT TCT GGG TGM CCA AAR AAT CA	Annealing	48˚C	00:30	35 ↺	
				Elongation	72˚C	01:30		
				Finalization	72˚C	03:00		
	Earthworm	Lum-ND2-322F or	TGT CAT YTC TGA TAC CCR TCA GT	Initial denaturation	94˚C	03:00		This study
	ND2	370F	TGC CTD ATY YTA TCN WCM TG	Denaturation	94˚C	00:30		
		Lum-COI-14R	CCA ATR TCT TTG TGG ATT WGT TGA GT	Annealing	43˚C	00:30	35 ↺	
				Elongation	72˚C	01:00		
				Finalization	72˚C	03:00		
	Verminephrobacter	VrpoB-43F	TTC GGC ASC CGC GAC AGC GYG C	Initial denaturation	94˚C	03:00		This study
	VrpoB	VrpoB-1430R	GCC ARR CCG GTG CGG TAC TGG TT	Denaturation	94˚C	00:30		
				Annealing	65˚C	00:30	30 ↺	
				Elongation	72˚C	01:30		
				Finalization	72˚C	03:00		
	Flexibacter-like	FrpoB-1905F	TGC YAA CCG YGC CYT GAT GG	Initial denaturation	94˚C	03:00		This study
	FrpoB	FrpoB-3213R	TGR CGW CCG GCC ATY TTA TC	Denaturation	94˚C	00:30		
				Annealing	60˚C	00:30	30 ↺	
				Elongation	72˚C	01:30		
				Finalization	72˚C	03:00		
	Flexibacter-like	Flexi-145F	GGY ATA GCT CGG GGA AAC	Initial denaturation	94˚C	03:00		Davidson et al., 2010
	16S rRNA gene	CF319aR	TGG TCC GTG TCT CAG TAC	Denaturation	94˚C	00:30		Manz et al., 1996
				Annealing	55˚C	00:30	30 ↺	
				Elongation	72˚C	00:30		
				Finalization	72˚C	03:00		
	Flexibacter-like	Flexi-145F	GGY ATA GCT CGG GGA AAC	Initial denaturation	94˚C	03:00		Davidson et al., 2010
	16S rRNA gene	1492R	GGT TAC CTT GTT ACG ACT T	Denaturation	94˚C	00:30		Turner et al., 1999
				Annealing	57˚C	00:30	30 ↺	
				Elongation	72˚C	01:30		
				Finalization	72˚C	03:00		
FISH probes	Eubacterial 16S rRNA	EUB338-I-II-III MIX-FAM	GCT GCC TCC CGT AGG AGT<br>GCA GCC ACC CGT AGG TGT<br>GCT GCC ACC CGT AGG TGT					Daims et al., 1999
	Verminephrobacter 16S rRNA	LSB145-CY5	GGG ATA ACG GAG CGA AAG					Schweitzer et al., 2001
	Flexibacter-like 16S rRNA	FLX226-CY3	ATC AGG CGC ATG CTT ATC C					This study
