# Supplementary material for: Evolution of the tripartite symbiosis between earthworms, Verminephrobacter and Flexibacter-like bacteria
Source: Front Microbiol. 2015 May 27;6:529. doi: 10.3389/fmicb.2015.00529 (PMC4445045; doi:10.3389/fmicb.2015.00529)

Supplementary Figure S1:

Bayesian inference of *Verminephrobacter* 16S rRNA gene sequences. Circles on nodes show posterior probabilities according to the legend. Colored boxes highligt groups of sequences from the same or from closely related host species. Vertical yellow lines show host species only colonized by *Verminephrobacter*. Vertical red lines show host species also colonized by *Ca*. Nephrothrix. Numbers on nodes are discussed in the text. All Verminephrobacter sequences are from host species from the family Lumbricidae.

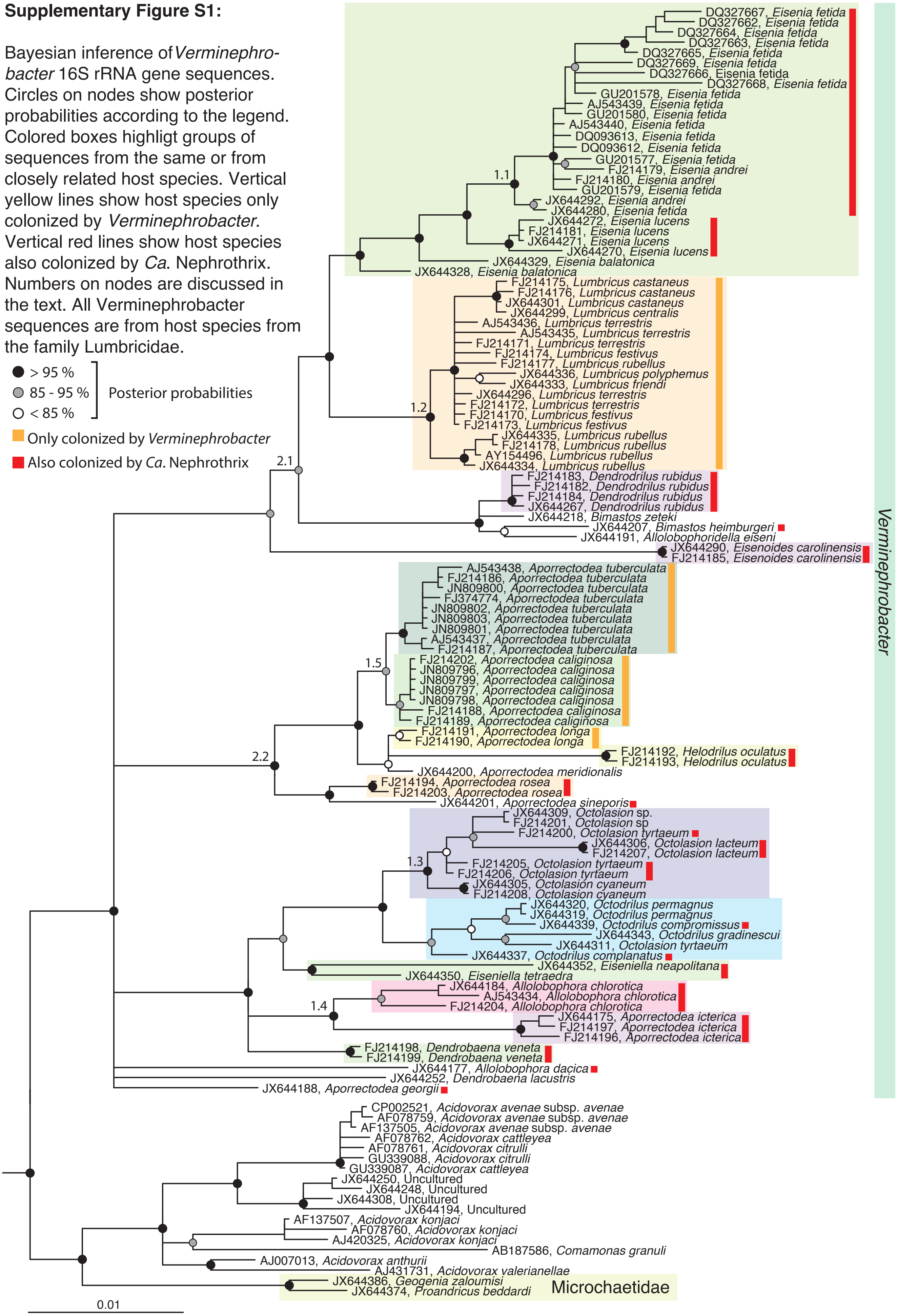

Supplement: Supplementary file 3 [file Image1.PDF]
